# Supplementary material for: Incorporating Robustness to Imaging Physics into Radiomic Feature Selection for Breast Cancer Risk Estimation
Source: Cancers (Basel). 2021 Nov 1;13(21):5497. doi: 10.3390/cancers13215497 (PMC8582675; doi:10.3390/cancers13215497)
Supplement: Supplementary file 1 [file cancers-13-05497-s001.zip › cancers-1328303-supplementary.pdf]

Supplementary Materials: Incorporating Robustness to Imaging Physics into Radiomic Feature Selection for Breast Cancer Risk Estimation

Raymond J. Acciavatti, Eric A. Cohen, Omid Haji Maghsoudi, Aimilia Gastounioti, Lauren Pantalone, Meng-Kang Hsieh, Emily F. Conant, Christopher G. Scott, Stacey J. Winham, Karla Kerlikowske, Celine Vachon, Andrew D. A. Maidment and Despina Kontos

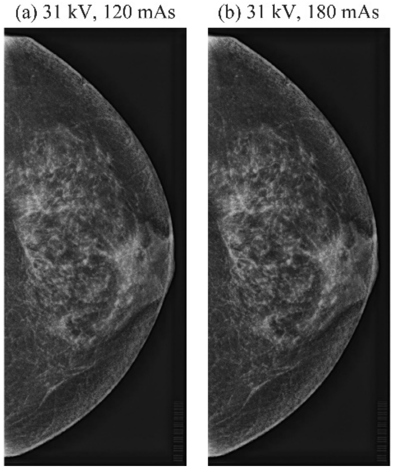

Figure S1. Example DM acquisitions of Gammex 169 phantom at two technique settings, illustrated here with processed ("FOR PRESENTATION") images, though the radiomic feature calculations were performed with raw ("FOR PROCESSING") images.

Table S1. Auto-time mAs setting at each kV for Gammex 169 phantom using W/Rh target/filter combination (data from Proc. SPIE 11314, Medical Imaging 2020: Computer-Aided Diagnosis, 113140W (16 March 2020); https://doi.org/10.1117/12.2549163).

kV: X-Ray Energy	mAs: Auto-Time
27	180
28	160
29	140
30	120
31	95
32	80
33	70
34	62
35	52

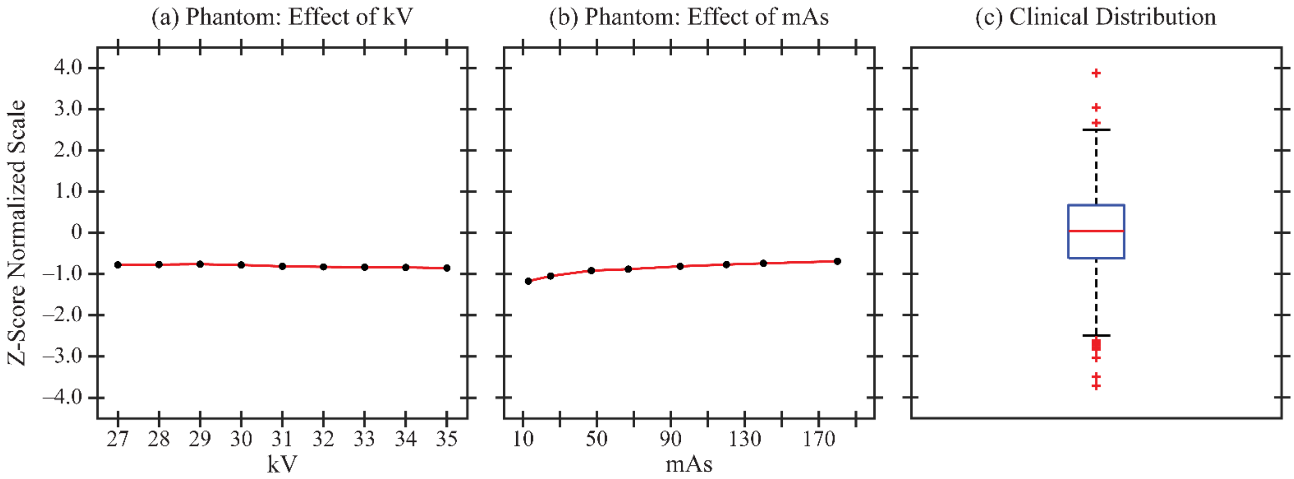

Figure S2. (**a**) Trends across kV for a robust feature (a Gabor Wavelet feature for which CMV = 0.11), with mAs at each kV given by Table S1 and each data point determined by the average of two acquisitions of the phantom, (**b**) Trends across mAs at a fixed kV (31 kV) for the same feature, with each data point determined by the average of two acquisitions of the phantom, (**c**) Distribution of feature values, used for Z-score normalization in all three subplots, derived from the subpopulation of women in Table 1 with thickness in [40, 60] mm.

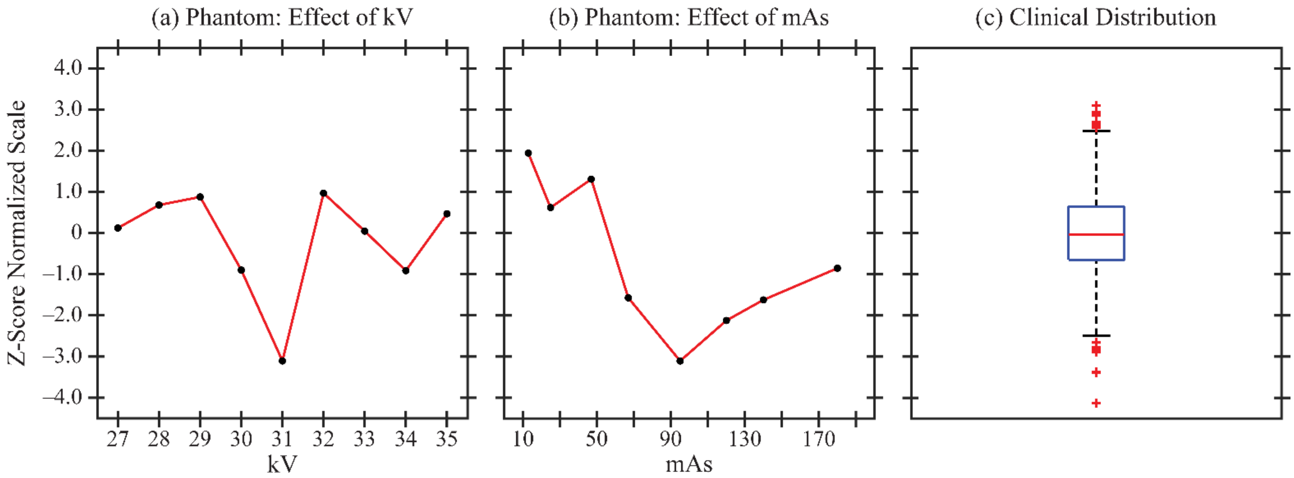

Figure S3. (**a**) Trends across kV for a nonrobust feature (a Laws feature for which CMV = 1.43), with mAs at each kV given by Table S1 and each data point determined by the average of two acquisitions of the phantom, (**b**) Trends across mAs at a fixed kV (31 kV) for the same feature, with each data point determined by the average of two acquisitions of the phantom, (**c**) Distribution of feature values, used for Z-score normalization in all three subplots, derived from the subpopulation of women in Table 1 with thickness in [40, 60] mm.

Table S2. Range of mAs values seen clinically in women with compressed breast thickness in [40, 60] mm; i.e., ± 10 mm relative to the phantom thickness (adapted from Proc. SPIE 11314, Medical Imaging 2020: Computer-Aided Diagnosis, 113140W (16 March 2020); https://doi.org/10.1117/12.2549163).

kV: X-Ray Energy	Clinical mAs Range for Breasts with Thickness in [40, 60] mm
28	[56, 228]
29	[63, 277]
30	[81, 386]
31	[102, 311]

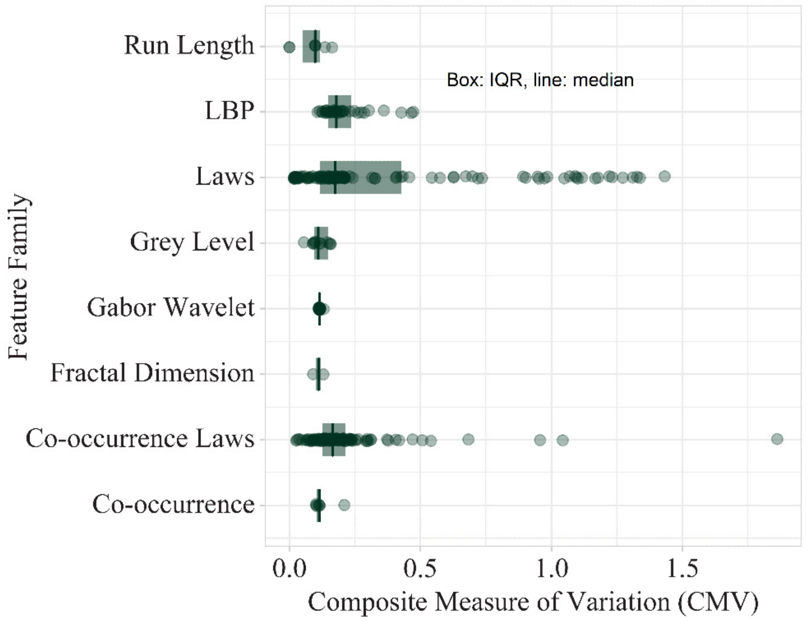

Figure S4. CMV results by feature family, with dots indicating individual features, shaded box the IQR, and crossbar the median value; there is no obvious association between family and feature robustness, with some families yielding very similar CMV for all features and some families spanning quite a range.

Table S3. Feature robustness quartile versus feature family (only features retained for case-control regression).

Feature Family: Number of Features	Number of Features in Robustness Class A	Number of Features in Robustness Class B	Number of Features in Robustness Class C	Number of Features in Robustness Class D
**Co-occurrence: 4**	3	0	1	0
**Co-occurrence Laws: 44**	10	15	14	5
**Fractal Dimension: 1**	1	0	0	0
**Gabor Wavelet: 0**	0	0	0	0
**Grey Level: 8**	4	4	0	0
**Laws: 39**	9	3	6	21
**LBP: 10**	0	3	6	1
**Run Length: 2**	0	2	0	0
